# Association of +67 G/A and -426 T/C Polymorphism in Eotaxin (CCL11) Gene with Psoriasis Phenotypes

**DOI:** 10.3390/genes16030288

**Published:** 2025-02-27

**Authors:** Vladimír Vašků, Adam Fiala, Anna Vašků

**Affiliations:** 1 1st Department of Dermatovenereology, St. Ann’s Faculty Hospital, Faculty of Medicine, Masaryk University, 602 00 Brno, Czech Republic; vladimir.vasku@fnusa.cz; 2 Psychiatrie Brno, Martinkova 253/7, Černá Pole, 612 00 Brno, Czech Republic; adam.fiala.md@gmail.com; 3 Department of Pathophysiology, Faculty of Medicine, Masaryk University, 625 00 Brno, Czech Republic

**Keywords:** psoriasis, CCL11, polymorphism

## Abstract

**Background/Objectives**: Several gene targets were identified for psoriasis. Some are currently being explored as potential therapeutic targets, including CCL11. Our task was to prove a possible association of single-nucleotide polymorphisms +67 G/A and -426 T/C in the eotaxin gene (CCL11, 17q 21.3) with the development and clinical aspects of psoriasis as an immune-based dermatological disease and evaluate its relationship to potential comorbidities. **Material and Methods**: In total, 460 patients with psoriasis were included in the case–control and genotype–phenotype study together with 167 control persons of similar age and sex distributions without a personal and/or family history of chronic disease of the skin. Two eotaxin gene polymorphisms were detected from isolated DNA via standard PCR, restriction analysis methods, and horizontal electrophoresis. **Results**: No significant case–control differences in the frequency of the CCL11 genotype in both polymorphisms were observed. In polymorphism +67 G/A, a significant increase in the AA genotype in patients with psoriasis guttata compared to plaque psoriasis was found (*p* = 0.006). A significant association of the A allele in psoriatic patients with a personal history of allergy was found (*p* = 0.02). The A alle was also significantly associated with a family history of psoriasis (*p* = 0.00008). In men, a higher risk of a delayed start of psoriasis (later than 40 years) associated with the T allele of -426 T/C polymorphism (*p* = 0.0007) was found. When double genotypes of both polymorphisms were evaluated, we observed significant differences in double genotype distribution between men with and without a family history of allergy (Pdg = 0.0005) and between those with and without affected siblings (Pdg = 0.03). In women with psoriasis, a higher risk of the TT genotype of -426 T/C polymorphism in patients with a personal history of diabetes (*p* = 0.001) as well as in patients with both a personal history of cardiovascular disease and diabetes (*p* = 0.00005) was proved. When double genotypes of both polymorphisms were evaluated, the significance of double genotype difference between those with and without personal history of diabetes was very high (Pdg = 0.0002). Similarly, the significance of the double genotype difference between those with and without personal history of cardiovascular diseases and diabetes was very high (Pdg = 0.000001). **Conclusions**: CCL11 is considered one of the basic chemokines responsible for the origin and development of immune-based reactions. Based on our results, we suggest that the +67 G/A CCL11 polymorphism should be considered as a gene modulator of psoriasis in specific subgroups of patients.

## 1. Introduction

Psoriasis is a complex, immune-mediated, chronic inflammatory disease of the skin. It involves genetic susceptibility, environmental triggers, and immune dysregulation [1]. Individuals with psoriasis are also at an increased risk of other co-morbidities, including psoriatic arthritis (PsA); metabolic syndrome or components of the syndrome; cardiovascular disorders; and several other diseases, such as anxiety and depression, non-alcoholic fatty liver disease, Crohn’s disease, and lymphoma [2].

Dendritic cells, T_H_17 cells, and keratinocytes seem to constitute the main pathogenic pattern in psoriasis. T-cell differentiation toward T_H_17 cells is stimulated by TNF-α and IL-23 produced by dendritic cells. Th17 cells produce key psoriatic cytokines IL-17, IFN-γ, and IL-22. Their activity results in skin inflammation and the activation and hyperproliferation of keratinocytes. In addition, other cells and signaling pathways are implicated in the pathogenesis of psoriasis, including T_H_9 cells, T_H_22 cells, CD8^+^ cytotoxic cells, neutrophils, γδ T cells, and cytokines and chemokines secreted by them [3]. IL-17 in psoriatic skin upregulates chemokines CCL2, CCL7, CCL20, and CXCL10; CXCL9 and CXCL10 were found to be upregulated even in non-lesional skin of psoriatic patients [3].

As explained, the main characteristics of psoriatic skin are hyperproliferation of keratinocytes and lesion infiltration by immune cells [4]. Genetic/epigenetic predisposition to psoriasis supports this feature against opposite (anti-proliferative, anti-inflammatory) mechanisms. Regarding the hyperproliferation of keratinocytes, tissue remodeling is necessary; some abnormal aspects of the extracellular matrix must contribute to the clinical manifestation of the disease. Thus, the development of psoriatic lesions seems to be regulated by cytokines, chemokines proteases, and their inhibitors [1,5].

When comparing psoriasis with other common chronic inflammatory skin diseases such as atopic dermatitis (AD), it is evident that they have common features (chronic inflammation and skin barrier dysfunction) but include different dysregulation of the immune system. Although psoriasis is centered on type 17 responses, AD is type 2 and type 22-skewed, with common type 1 polarization developing in chronic lesions [6]. Three potentially pathogenic chemokine pathways have been identified in atopic dermatitis (CCL17/CCL22-CCR4, CCL27-CCR10, and CX3CL1-CX3CR1), and two in psoriasis (CCL20-CCR6 and CCL17/CCL22-CCR4), where one of them seems to be common [7].

CCL11 (eotaxin-1) is produced by epithelial cells, smooth muscle cells, fibroblasts, chondrocytes, and macrophages. Using receptor CCR3 on the cell surface, it binds to Th2 lymphocytes, eosinophils, basophils, and mast cells to modify their action. As a pathophysiological effect, participation in many allergic reactions may develop. Moving through the hematoencephalic barrier and/or produced by astrocytes, microglia, or cells of the choroid plexus in the brain, it can modify the action of astrocytes and microglia to produce cytokines and ROS, which can lead to decreased adult neurogenesis, neuronal damage, and the aging of neurons [8]. CCL11protein was found to be upregulated in patients with AD, not with psoriasis [9]. Based on all these findings, CCL11 is considered a key modulator of allergic reactions and, therefore, influencing eotaxin seems to be promising in therapy for various immune-based diseases [10,11]. Interestingly, when the potential therapeutic target for psoriasis and psoriatic arthritis (PsA) was analyzed via priority index calculation (based on GWAS), CCL11 was identified as a target for PsA therapy [11].

Our task was to prove a possible association of single-nucleotide polymorphisms +67 G/A and -426 T/C in the eotaxin gene (CCL11, 17q 21.3) with the development and clinical aspects of psoriasis as an immune-based dermatological disease.

## 2. Material and Methods

Subjects: This study included patients with psoriasis, 253 men (age 60 ± 17 years) and 207 women (age 60 ± 18), and 167 control persons (age 55 ± 5 years). Control patients were obtained from the database of healthy non-related persons from general practitioners obtained at the Department of Pathophysiology: a group of similar age and sex distributions without personal or family history of chronic disease of the skin was selected.

All evaluated persons were from the same population of Central European origin with permanent residence in the South Moravian region of the Czech Republic. All psoriasis patients were diagnosed and treated in accordance with criteria set up by American Academy of Dermatology at the First Department of Dermatovenereology at St. Anne’s University Hospital Brno, Czech Republic. Four clinical subtypes of psoriasis were distinguished among the patients, i.e., plaque psoriasis (n = 339), pustular psoriasis (n = 22), psoriasis arthropathica (n = 17), and psoriasis guttata and nummularis (n = 82). In psoriasis patients, 29% of men and 33% of women had a positive family history of psoriasis in first-degree relatives. Frequencies of complex comorbidities of psoriasis patients are presented in Table 1.

The patients were diagnosed and treated either topically with topical corticosteroids, tar, vitamin D3 analogs, with phototherapy and photochemotherapy (SUP and PUVA), or with systemic acitretin and interferon γ treatment combinations.

This study was approved by the Committee for Ethics of Medical Experiments on Human Subjects, Faculty of Medicine, Masaryk University, Brno, and was performed in adherence to the Declaration of Helsinki Guidelines. Participants gave their written informed consents, which have been archived.

Genotyping:

Two CCL11 gene polymorphisms were detected from isolated DNA via standard PCR, restriction analysis methods, and horizontal electrophoresis in the laboratories of the Department of Pathophysiology, where the methods were optimized and performed.

Genomic DNA was purified from peripheral blood leukocytes via the standard method using phenol–chloroform extraction and the proteinase K digestion of cells. We investigated a single-nucleotide polymorphism (SNP) within eotaxin promoter and one SNP in exon 1 of eotaxin gene: C>T at position −426 (rs16969415) and SNP 67 G>A (rs1129844). SNPs were analyzed using PCR followed by restriction enzyme analysis. Each PCR was carried out in the volume of 25 μL using Taq polymerase. All restriction enzyme analyses were carried out in a volume of 10 μL. Restriction analysis of C>T at position −426 was performed using TaqI restriction enzyme. The length of the fragments was 204 and 41 bp for -426C and 245 bp for -426T. The genotypes were detected via electrophoresis.

The SNP 67 G>A (rs112844) was determined using PCR method and restriction analysis using restriction endonuclease BsrI. Fragments were detected via electrophoresis on 2% agarose gel. More detailed description of genotyping methods is presented in our older articles [12].

## 3. Statistical Analysis

The distributions of genotype and allelic frequencies and their differences were calculated using χ^2^ tests. The consistency of genotype frequencies with the Hardy–Weinberg equilibrium was tested using a χ^2^ test. The odds ratio (OR) and 95% confidence interval were calculated to estimate the risks related to detected polymorphisms. To calculate the significance of the OR, Fisher’s exact test was used. Clinical Calculator 1—Vassar Stats was applied for the calculation of the sensitivity and specificity of results. The program package Statistica v. 14.0 (Statsoft Inc., Tulsa, OK, USA) was used.

## 4. Results

No significant case–control differences in the frequency of the CCL11 genotype in both polymorphisms were observed (Table 2).

In polymorphism +67 G/A, a significant increase in the AA genotype in patients with psoriasis guttata compared to plaque psoriasis was found (odds ratio = 5; 95% confidence interval = 1.42–20.6, *p* = 0.006, sensitivity = 0.061, specificity = 0.988, power test = 0.882; Table 3). This trend is more pronounced in men (OR = 8.68; 95% CI = 1.54–49.03, *p* = 0.004, sensitivity = 0.09, specificity = 0.989, power test = 0.628) than in women when evaluated separately.

In polymorphism +67 G/A, the A allele was highly significantly associated with a family history of allergy (28% of patients with a family history of allergy, OR = 2.12, 95% confidential interval = 1.45–3.09, *p* = 0.00008, sensitivity = 0.257, specificity = 0.860, power test = 0.404). Further, a significant association of the A allele with a personal history of allergy was found in psoriatic patients (OR = 1.63, 95% confidential interval = 1.07–2.49, *p* = 0.02, sensitivity = 0.220, specificity = 0.853, power test = 0.546).

When both polymorphism associations were tested together (double genotype—nine possible combinations), the GGCC genotype was also associated with a family history of allergy (OR = 1.72, 95% CI = 1.14–2.59, *p* = 0.01, sensitivity = 0.488, specificity = 0.650, power test = 0.898; Table 4).

In polymorphism -426 T/C, there was a significant association of TT genotype with a personal history of both cardiovascular disease and diabetes (OR = 12.2, 95% confidential interval = 1.06–140.8, *p* = 0.061, sensitivity = 0.05, specificity = 0.995, power test = 0.356).

In men, a higher risk of delayed onset of psoriasis (later than in 40 years) associated with the T allele of the -426 T/C polymorphism (OR = 4; 95% confidential interval = 1.7–9.62, *p* = 0.0007, sensitivity = 0.100, specificity = 0.973, power test = 0.764) was found.

When double genotypes of both polymorphisms were evaluated, we observed significant differences in the double genotype distribution between men with and without a family history of allergy (Pdg = 0.0005) and between those with and without siblings (brothers and sisters) with psoriasis (Pdg = 0.03).

In women with psoriasis, a higher risk of the TT genotype of the -426 T/C polymorphism in patients with a personal history of diabetes (OR = 25, 95% CI = 1.45–443, *p* = 0.001, sensitivity = 0.111, specificity = 0.995, power test = 0.448) and in patients with both a personal history of cardiovascular disease and diabetes (OR = 41, 95% CI = 2.24–757, *p* = 0.00005, sensitivity = 0.167, specificity = 0.995, power test = 0.516) was proved (Table 5).

When double genotypes of both polymorphisms were evaluated, the significance of the double genotype difference between those with and without a personal history of diabetes was very high (Pdg = 0.0002). Similarly, the significance of double genotype differences between those with and without both a personal history of cardiovascular diseases and diabetes was very high (Pdg = 0.000001, Table 6).

## 5. Discussion

No similar study analyzing the associations of CCL11 gene variability with psoriasis and some phenotypes of comorbidities was observed by us in the literature. On the other side, CCR3 (receptor for CCL11), together with seven other genes (*SIGLEC8*, *IL5RA*, *RNASE2*, *CPA3*, *GATA2*, *c-KIT*, and *PRSS33*), was identified as a potential diagnostic biomarker and therapeutic target for psoriasis using highly sophisticated Weighted Gene Co-expression Network Analysis (WGCNA) [13].

Biological pathways implicated in psoriasis via genome-wide association studies (GWAS) do not include chemokines genes directly (IL23/IL 17 axis, antigen presentation, skin barrier, NF-κB signaling, and type I interferon signaling) [14]. Surprisingly, no significant association between CCL11 gene variability and atopic dermatitis was found in a meta-analysis [15].

A significant association between the A allele in the CCL11 67 G/A single-nucleotide polymorphism and a lower survival rate in patients with coronary artery disease was found in a Central European (Czech) population [12]. CCL11 promotor polymorphisms (including -426 T/C in three haplotypes) were found to be associated with an increased risk of the development of schizophrenia in the Korean population [16]. CCL11 is known to be a potent agonist for chemokine receptor CCR3 that can attract eosinophils at sites of inflammation [17]. Interaction among single-nucleotide polymorphisms (SNPs) in CCR3 and eotaxin genes is associated with immune-based diseases [18].

Gaspar et al. [19] tried to identify chemokine-mediated communication pathways regulating tissue remodeling during atopic skin and psoriasis inflammation and investigate the function of chemokine receptor CCR3 on human dermal fibroblasts. Corresponding ligand CCL26 to the chemokine receptor CCR3 demonstrated significant and specific atopic upregulation when compared to psoriatic skin inflammation. In dermal fibroblasts, CCL26 induced CCR3 signaling, resulting in intracellular Ca (2+) mobilization, as well as enhanced fibroblast migration and repair capacity, but no proliferation.

Recently, there has been a strong need to identify reproducible experimental approaches that closely mimic their molecular phenotype and can be used for the evaluation of new drug candidates in human pre-clinical studies. Diphencyprone (DPCP) was observed to induce the strongest immune responses across all pathways and modeled barrier defects characteristic of AD and psoriasis. Ni induced all immune pathways but to a limited extent compared to DPCP. Purified protein derivative (PD) predominantly induced Th1 and Th17, while dust mite (DM) predominantly induced Th2 and Th17/Th22 inflammation. Thus, it was shown that contact hypersensitivity reactions may be suitable experimental approaches to model various immune and/or barrier alterations seen in the skin of patients with inflammatory skin diseases. According to the results of this study, the Th2 axis showed significantly increased expression across all groups, with the strongest induction seen with DPCP, followed by Ni and DM for IL-13/IL-31/IL-4R/CCL11/CCL13/CCL17/CCL18/CCL22. Until now, a molecular study that compares the simultaneous ability of different sensitizers to model various inflammatory skin diseases is lacking [20]. This could be a very helpful approach for us to compare different functional characteristics of the skin barrier in psoriasis carriers of different genotypes of candidate genes.

In conclusion, similar genetic characteristics may contribute to the data assembly of genetic predisposition to psoriasis candidate genes, which could lead to therapy improvement based on time-proved individual pharmacogenetic aspects detected in psoriasis patients. In our case, the situation is complicated by gene association with family and/or a personal history of complex diseases, which need stronger interdisciplinary cooperation to achieve the best modus of patient therapy. As usual, other similar studies performed on other populations are necessary for a final discussion of our results.

## Figures and Tables

**Table 1:** Comorbidity frequencies in patients with psoriasis.

Patients with Psoriasis—Personal History (N = 460)	Men (N = 253)	Women (N = 207)
Cardiovascular diseases		
psoriasis vulgaris chronical (N = 339)	34%	32%
psoriasis pustulosa (N = 22)	30%	42%
psoriasis arthropathica (N = 17)	36%	22%
psoriasis guttata and numularis (N = 82)	20%	13%
Diabetes mellitus		
psoriasis vulgaris chronica	10%	5%
psoriasis pustulosa	0	0
psoriasis arthropathica	0	0
psoriasis guttata and numularis	5%	4%
Allergy		
psoriasis vulgaris chronica	13%	19%
psoriasis pustulosa	10%	33%
psoriasis arthropathica	18%	11%
psoriasis guttata and numularis	22%	28%
Tumors		
psoriasis vulgaris chronica	9%	14%
psoriasis pustulosa	0	8%
psoriasis arthropathica	9%	33%
psoriasis guttata and numularis	3%	7%

**Table 2:** Case–control study results for +67 G/A and -426 T/C polymorphisms in CCL11 gene.

***+67 G/A*** ***CCL11***	**GG**	**GA**	**AA**	**Minor Allele Frequency**	***p*-Value**	**Total**
Patients	322 (0.70)	129 (0.28)	9 (0.02)	0.160	Pg = 0.289 Pa = 0.802	460
Controls	119 (0.71)	46 (0.28)	2 (0.01)	0.150		167
***-426 T/C***	**CC**	**CT**	**TT**	**Minor allele** **Frequency**		**Total**
Patients	416 (0.90)	41 (0.09)	3 (0.01)	0.051	Pg = 0.145 Pa = 0.507	460
Controls	146 (0.87)	19 (0.12)	2 (0.01)	0.069		167

Pg = probability of a difference in genotype distribution, Pa = probability of a difference in allele frequency.

**Table 3:** Type of psoriasis and +67 A/G CC11 polymorphism.

Type of Psoriasis		+67 CCL11 GA	+67 CCL11 AA	Minor Allele Frequency	Total
1 *	236 (0.70)	97 (0.29)	4 (0.01)		337
2	16 (0.73)	6 (0.27)	0 (0)		22
3	13 (0.76)	4 (0.24)	0 (0)		17
4	56 (0.68)	21 (0.26)	5 (0.06)		82

1 *—psoriasis vulgaris chronica, 2—psoriasis pustulosa, 3—psoriasis arthropathica, 4—psoriasis guttata and numularis.

**Table 4:** Family history of allergy in psoriatic patients and double genotypes in CCL11 gene polymorphisms.

Family History of Allergy	GGCC	GGCT	GGTT	GACC	GACT	AACC	Total
0 (0.72)	215 (0.65)	28 (0.08)	1 (0.003)	80 (0.24)	1 (0.003)	6 (0.02)	331
1 (0.28)	67 (0.52)	9 (0.07)	2 (0.02)	45 (0.35)	3 (0.03)	3 (0.03)	129

**Table 5:** Positive personal history of cardiovascular diseases and diabetes and -426 T/C CCL11 polymorphism genotype distribution in women.

PHCV + DM *	-426 T/C CCL11 CC	-426 T/C CCL11 CT	-426 T/C CCL11 TT	Total
0 (0.97)	184 (0.88)	23 (0.12)	0 (0)	207
1 (0.03)	5 (0.83)	0 (0)	1 (0.17)	6

PHCV + DM *—personal history of cardiovascular diseases and diabetes mellitus.

**Table 6:** Positive personal history of cardiovascular diseases and diabetes and double genotypes in CCL11 gene polymorphisms in women.

PHCV + DM *	GGCC	GGCT	GGTT	GACC	GACT	AACC	Total
0 (0.97)	116 (0.56)	20 (0.10)	0 (0)	65 (0.31)	3 (0.01)	3 (0.01)	207
1 (0.03)	4 (0.67)	0 (0)	1 (0.17)	1 (0.17)	0 (0)	0 (0)	6

PHCV + DM *—personal history of cardiovascular diseases and diabetes mellitus.

## Data Availability

The original contributions presented in the study are included in the article, further inquiries can be directed to the corresponding author.
